# HLA-G and HLA-E Immune Checkpoints Are Widely Expressed in Ewing Sarcoma but Have Limited Functional Impact on the Effector Functions of Antigen-Specific CAR T Cells

**DOI:** 10.3390/cancers13122857

**Published:** 2021-06-08

**Authors:** Bianca Altvater, Sareetha Kailayangiri, Lina F. Pérez Lanuza, Katja Urban, Lea Greune, Maike Flügge, Jutta Meltzer, Nicole Farwick, Simone König, Dennis Görlich, Wolfgang Hartmann, Claudia Rossig

**Affiliations:** 1 Department of Pediatric Hematology and Oncology, University Children’s Hospital Muenster, 48149 Muenster, Germany; bianca.altvater@ukmuenster.de (B.A.); sareetha.kailayangiri@ukmuenster.de (S.K.); linafranziska.perezlanuza@ukmuenster.de (L.F.P.L.); katja.urban@ukmuenster.de (K.U.); lea.greune@ukmuenster.de (L.G.); maikef@miltenyi.com (M.F.); jutta.meltzer@ukmuenster.de (J.M.); nicole.farwick@ukmuenster.de (N.F.); 2 Core Unit Proteomics, Interdisciplinary Center for Clinical Research, 48149 Muenster, Germany; koenigs@uni-muenster.de; 3 Institute of Biostatistics and Clinical Research, University of Muenster, 48149 Muenster, Germany; dennis.goerlich@ukmuenster.de; 4 Gerhard-Domagk-Institute of Pathology, University of Muenster, 48149 Muenster, Germany; wolfgang.hartmann@ukmuenster.de; 5 Cells-in-Motion Cluster of Excellence (EXC 1003-CiM), University of Muenster, 48149 Muenster, Germany

**Keywords:** cellular immunotherapy, checkpoint inhibitors, Ewing sarcoma, HLA-G, HLA-E

## Abstract

**Simple Summary:**

Solid cancers can effectively counteract immune attack by inhibitory checkpoints in the tumor microenvironment. Blockade of relevant immune checkpoints could be a useful tool for enhancing the efficacy of antitumor T cell therapies. Here, we studied the capacity of two nonclassical HLA molecules with known immunosuppressive function, HLA-G and HLA-E, to prevent antigen-specific immune effector functions of gene-engineered T cells against Ewing sarcoma. Inflammatory conditions and interactions of Ewing sarcoma cells with antitumor T cells reliably induced upregulation of the two molecules on the tumor cells. Moreover, as previously shown for HLA-G, HLA-E was detected in a high proportion of human Ewing sarcoma biopsies. However, artificial expression of either of the two molecules on Ewing sarcoma cells failed to reduce cytolytic and activation responses of antigen-specific T cells. We conclude that blockade of HLA-G and HLA-E immune checkpoints is not a promising strategy for enhancing T cell therapies in Ewing sarcoma.

**Abstract:**

Immune-inhibitory barriers in the tumor microenvironment of solid cancers counteract effective T cell therapies. Based on our finding that Ewing sarcomas (EwS) respond to chimeric antigen receptor (CAR) gene-modified effector cells through upregulation of human leukocyte antigen G (HLA-G), we hypothesized that nonclassical HLA molecules, HLA-G and HLA-E, contribute to immune escape of EwS. Here, we demonstrate that HLA-G isotype G1 expression on EwS cells does not directly impair cytolysis by G_D2_-specific CAR T cells (CART), whereas HLA-G1 on myeloid bystander cells reduces CART degranulation responses against EwS cells. HLA-E was induced in EwS cells by IFN-γ stimulation in vitro and by G_D2_-specific CART treatment in vivo and was detected on tumor cells or infiltrating myeloid cells in a majority of human EwS biopsies. Interaction of HLA-E-positive EwS cells with G_D2_-specific CART induced upregulation of HLA-E receptor NKG2A. However, HLA-E expressed by EwS tumor cells or by myeloid bystander cells both failed to reduce antitumor effector functions of CART. We conclude that non-classical HLA molecules are expressed in EwS under inflammatory conditions, but have limited functional impact on antigen-specific T cells, arguing against a relevant therapeutic benefit from combining CART therapy with HLA-G or HLA-E checkpoint blockade in this cancer.

## 1. Introduction

Ewing sarcoma is an aggressive bone and soft tissue cancer predominantly arising in children and adolescents. Intensive multimodality therapy only cures a proportion of patients [1,2]. In hematological cancers, adoptive immunotherapy with T cells engineered to express chimeric antigen receptors (CARs) has been successfully developed as a novel therapeutic option for patients with refractory cancers [3,4]. A potential CAR T cell (CART) target in EwS is the ganglioside antigen G_D2_, which can be highly overexpressed on the tumor cell surface [5]. However, while T cells and NK cells that are gene-modified to express G_D2_-specific CARs effectively interact with EwS cells in vitro, adoptive transfer in preclinical in vivo models so far failed to eradicate the disease, despite a consistent and high expression of the target antigen [6,7,8].

One potential explanation for the lack of activity of T cell therapeutics in EwS and other solid cancers is the presence of immune-inhibitory components in the tumor microenvironment (TME), which prevent the infiltration of T cells and other immune effector cells and tolerize the cells against tumor targets [9]. A powerful inhibitory immune checkpoint is PD-L1 engaging the PD-1 receptor on T cells, to regulate effector T cell responses in tissues [10]. Therapeutic antibody blockade of PD-1 or PD-L1 has been successful in patients with various cancers but is ineffective in others, including EwS [11,12]. To enable effective CART therapy of EwS, relevant barriers in the TME must be identified and co-targeted. 

Candidate negative regulators of T cell function in EwS are the non-classical HLA tolerogenic molecules HLA-G and HLA-E, both strong local inhibitors of cellular immune responses [13,14]. Unlike classical HLA molecules, HLA-G and -E have low genetic diversity and play no role in peptide presentation to T cell receptors. HLA-G has a limited polymorphism with only 7 isoforms (HLA-G1 to G7), which interact with three inhibitory receptors expressed on T cells, NK cells and myeloid cells and directly or indirectly reduce effector cell proliferation and functions [15,16]. HLA-E negatively affects the cytotoxic function of both CD8+ T cells and NK cells by engaging the inhibitory receptor NKG2A/CD94 [14,17]. Both HLA-G and HLA-E are commonly overexpressed on tumor cells, and expression is inducible by inflammatory cytokines [18,19,20,21,22,23]. Experimental evidence suggests important contributions of HLA-G and HLA-E to immune escape in many cancers [18,23,24,25]. Thus, both HLA-G and HLA-E are candidate immune-inhibitory checkpoints that could prevent antitumor immune responses. Towards a clinical strategy, an antibody antagonist of the HLA-E/NKG2A interaction was developed, which indeed potentiates antitumor immunity by T cells and NK cells, and thus represents an attractive combination partner for cellular immunotherapeutics [18].

In previous work in EwS, we found that tumor xenografts in response to adoptive therapy with G_D2_-redirected CAR NK cells or CART strongly upregulate the nonclassical MHC class I molecule HLA-G [6,26]. Moreover, we found that HLA-G is expressed in a relevant proportion of human pre-treatment EwS biopsies either on the tumor cells or on infiltrating lymphocytes and associated with the presence of infiltrating T cells [26]. HLA-E has not yet been studied in EwS.

Here, we pursued the hypothesis that HLA-G or HLA-E are key contributors to local immune suppression in EwS and are targetable immune checkpoints for more effective CART therapy. We used isoform-specific antibodies to identify the HLA-G isoform upregulated in EwS cells through exposure to inflammatory cytokines by Western Blot and by ELISA. Then, we investigated the capacity of HLA-G expressed either by G_D2_-positive EwS cells or by myeloid bystander cells to negatively affect antigen-specific activation and cytolytic responses of G_D2_-specific CART in in vitro coincubation experiments. We further studied expression of HLA-E in immune infiltrates of human EwS tissues and xenografts, through multicolor immunofluorescence analysis, then analyzed the functional consequences of HLA-E overexpression by EwS cells on G_D2_-specific CART.

## 2. Materials and Methods

**Cell lines and IFN-γ pretreatment.** The Ewing sarcoma cell lines A673 and TC-71 were obtained from DSMZ (Braunschweig, Germany), VH-64 was kindly provided by Frans van Valen’s laboratory at the Institute of Experimental Orthopedics of University of Muenster, Germany, and A4573 and TC-32 were a gift from the Children’s Hospital Los Angeles. Collagen-coated 25 cm^2^ tissue culture flasks were used to culture EwS cell lines in RPMI 1640 medium (Thermo Fisher Scientific, Dreeich, Germany) containing 10% heat-inactivated fetal calf serum (FCS; Thermo Fisher Scientific, Waltham, MA, USA) and 2 mM L-glutamine (PAA, Freiburg, Germany), at 37 °C and 5% CO_2_. The human leukemia cell line K562 and the human myeloid cell line THP-1 were purchased from DSMZ and cultured in uncoated tissue culture flasks. For some experiments, EwS cells, THP-1, and K562 cells were retrovirally transduced with the HLA-G1 gene (UniProtKB-P17693 Isoform 1), as previously described [6], or with HLA-G5 (UniProtKB-P17693 Isoform 5). THP-1 were retrovirally transduced with HLA-E (UniProtKB-P13747) and a combination of the G_D3_ synthase (GD3S, UniProtKB-Q92185) and the G_D2_ synthase (GD2S, UniProtKB-Q00973) genes, to enable the synthesis of ganglioside G_D2_. The identity of the cell lines was confirmed by short tandem repeat (STR) profiling. For IFN-γ cytokine pretreatment, EwS cell cultures at 30–50% confluence were stimulated with a medium containing 2000 U/mL IFN-γ for 48 h.

**Western Blot analysis.** Western blot analysis was performed as previously described [6]. In brief, anti-HLA-G antibody clone 4H84 (Exbio, Vestec, Czech Republic) or anti-HLA-G antibody clone 5A6G7 (Abcam, Cambridge, UK), each diluted 1:1000 in TBST (5% BSA), were used as primary antibodies, followed by detection with HRP-linked secondary antibody and enhanced chemiluminescence reagent. After stripping, β-Actin antibody (Cell Signaling) diluted 1:5.000 in TBST (5% BSA) was added to determine the equal protein loading. The original western blotting figures can be found in Figure S1.

**ELISA.** Protein lysates were generated as above and HLA-G expression was analyzed using a commercially available HLA-G kit (Exbio) for specific detection of HLA-G1 and 5 isoforms. Lysates from HLA-G1 and HLA-G5-transduced and wild-type K562 cells served as positive and negative controls, respectively. HLA-G (U/mL) was quantified by measurement of the incorporated calibrator.

**Flow cytometry analysis.** At least 100,000 tumor cells were stained with fluorescence-conjugated monoclonal antibodies (mAbs) against HLA-G (clone MEM/G9, Exbio), HLA-E (clone 3D12, Biolegend, Fell, Germany) and G_D2_ (clone 14.G2a, Biolegend), respectively, or the corresponding fluorescence-conjugated isotype controls (BioLegend). CART were stained with fluorescence-conjugated monoclonal antibodies against CD3 (clone SK7, Biolegend), CD8 (clone RPA-T8, Biolegend), CD94 (clone HP3D9, BD Pharmingen), CD85j (clone HP-F1, eBioscience, Frankfurt, Germany), NKG2A (clone 131411, R&D, Brooklyn Park, MN, USA) and the CAR-specific anti-idiotype antibody ganglidiomab, provided by H. Lode, Greifswald, Germany, and fluorescence-labeled with the Mix-n-Stain Kit (Sigma-Aldrich, Taufkirchen, Germany). Samples were fixed with 1% paraformaldehyde (PFA) and acquired directly or not later than 24 h after staining. For each sample, 10,000 cells within the respective gates were analyzed with FACS Diva 8.0, using FACS Celesta flow cytometer (BD Biosciences, Germany) and FlowJo version 10 (FlowJo, LLC, Ashland, OR, USA).

**CRISPR/Cas9 knockout.** The sgRNAs used to knock out the gene encoding HLA-G1 (UniProtKB-P17693 Isoform 1) were designed using the website http://crispr.mit.edu/ (accessed on 20 May 2021). The sgRNAs were cloned into the BsmBI sites of the lentiviral vector lentiCRISPR_v2 (Addgene 52961) [27]. The production of lentiviral supernatant in HEK293T cells was performed, as previously described [28]. HLA-G1 gene knockout was analyzed by Western Blot analysis, after one week of puromycin (6 µg/mL) selection. Single cell cloning was done by diluting sgRNA129 transduced A4573 cells to 0.5 and 1 cell per mL, and plating in 96-well plates with 200 µL per well with continuous puromycin selection. Viable cell cultures were picked after 3 to 4 weeks of culture and analyzed by PCR, after gDNA extraction with primers flanking the HLA-G α1 domain (HLA-GCRISPR-fw: ACTCCCATTAGGTGACAGGTTTTTA and HLA-GCRISPR-rev: ATCGTAGGCATACTGTTCATACCC). All clones with a PCR product different from the WT were sequenced (LGC) after cloning of the PCR product into a TOPO TA cloning vector (Thermo Fisher Scientific, Waltham, MA, USA). A4573 clone 16 was then analyzed by Western Blot and ELISA, as described above.

**CAR constructs and transduction of human T cells.** The G_D2_-specific CAR construct and the methods used for transduction of human T cells were previously described [6].

**Legendplex cytokine bead assay and MACSquant analysis.** For cytokine analysis, 1 × 10^5^ GD2-BBζ transduced T cells per well were seeded in a 96 well plate together with 1 × 10^5^ irradiated HLA-G1-transduced VH-64 cells per well, and incubated for 48 h at 37 °C and 5% CO_2_. Mock-transduced VH-64 cells were used as control. Supernatants were harvested and analyzed with the Legendplex Cytokine Bead Assay Human CD8/NK Panel (13-plex) from BioLegend, according to the manufacturer’s specifications. Samples were analyzed after acquisition on a MACSquant Flow Cytometer (Miltenyi Biotech, Bergisch Gladbach, Germany) with the LEGENDplex v8.0 Software (BioLegend, Fell, Germany).

**CD107a assay.** Target cells and effector cells were treated with 1 μL/mL Monensin (Biolegend) and co-cultured at a 1:1 ratio for 3 h at 37 °C, in the presence of CD107a BV510 (Biolegend). Then, the cells were washed and stained with CF488 fluorescence-labeled CAR anti-idiotype antibody ganglidiomab, APC-Fire750-labeled anti-CD3 (Biolegend) and either anti-CD85j-PE or anti-NKG2A-PE for 15 min in the dark. Cells were washed and analyzed by flow cytometry. To block HLA-G interactions, tumor cells were preincubated with 20 µg/mL of either 87G antibody or IgG2a isotype control for 15 min, prior to adding the T cells. At least 400 events were analyzed within the gates of either CD3+/CAR+/CD85j+ or CD3+/CAR+/NKG2A+ cells, respectively.

**Cytotoxicity assay.** To test the lytic activity of CART, a calcein acetyoxymethyl (AM) release assay was used, as previously described [28]. In brief, calcein-stained target cells were coincubated with CART for 4 h at an effector-to-target cell ratio of 40:1 or alone, and fluorescence was quantified using the microplate reader GloMax^®^ Discover (Promega, Walldorf, Germany).

**Patient material.** Tumor biopsies from EwS patients treated at University Children’s Hospital Muenster within the multicenter clinical studies EURO-E.W.I.N.G. 99 and EWING 2008 were included with the consent of patients or their legal guardians, in accordance with the Declaration of Helsinki.

**Conventional immunohistochemistry (IHC).** Formalin-fixed paraffin-embedded (FFPE) sections of human tumor biopsies or EwS xenografts were deparaffinized and manually stained with human HLA-E specific antibody (clone MEM-E/02, ExBio). HLA-E expression was analyzed for 40 min, after heat-induced antigen retrieval in a pressure cooker with citrate buffer (pH9), followed by blocking with serum-free blocking solution (Dako, Jena, Germany) for 1 h at RT. The slides were then incubated overnight at 4 °C with unconjugated HLA-E antibody, and 1:25 diluted in Antibody Diluent (Dako). Immunoreactions were visualized using a biotinylated polyclonal secondary antibody (BD Pharmingen) and Peroxidase/DAB detection. Slides were counterstained with hematoxylin and covered with Cytoseal (Thermo Fisher). Human tonsil tissue was used as positive control. The intensities of HLA-E antibody staining were defined by the pathologist, using a semiquantitative scoring system with three staining intensities (strong, weak, and negative).

**Multicolor fluorescence staining.** Multiplexed fluorescence staining was performed by the tyramide signal amplification (TSA) method using Opal fluorophores (Akoya Biosciences, Marlborough, MA, USA). Microwave treatment between each staining cycle was used to remove primary and secondary antibodies, while retaining the fluorescent signal. FFPE sections were deparaffinized in xylene, rehydrated, and washed in water, before staining with a self-designed 5-plex panel with antibodies, against the pan T cell marker CD3 (clone 26V6, Ventana, Tucson, AZ, USA), the pan-macrophage marker CD68 (clone KP-1, Ventana), HLA-G (clone 4H84, ExBio) or HLA-E (clone MEM-E/02, ExBio) and the EwS tumor cell marker CD99 (clone HCD99, Biolegend). To optimize the panel, we first selected optimal antibody clones by standard IHC staining, then linked them with opal fluorophores (Akoya Biosciences) and performed single-plex immunofluorescence staining. Finally, we optimized the sequences of staining with the individual antibodies and their concentrations as well as the individual fluorophore pairings. Immunofluorescence staining was performed manually with 4 staining cycles. First staining cycle—antigen retrieval (AR) with citrate buffer pH6, blocking with serum-free blocking solution (Dako) for 1 h at RT, incubation with anti-CD3 antibody (undiluted) overnight at 4 °C, incubation with secondary antibody (polymer HRP Ms+Rb, Akoya Bioscience) for 10 min at RT, then incubation with Opal 540 (1:50) for 10 min at RT. Second staining cycle—microwave treatment with citrate buffer pH6, blocking as above, incubation with anti-CD68 (undiluted) antibody overnight at 4 °C, incubation with secondary antibody as above, then incubation with Opal 520 (1:50) for 10 min at RT. Third staining cycle—microwave treatment and blocking as above, incubation with anti-HLA-G (1:100) or HLA-E (1:25) antibodies, respectively, in antibody diluent overnight at 4 °C, incubation with secondary antibody as above, then with Opal 570 (1:50) for 10 min at RT. Final staining cycle—microwave treatment and blocking as above, incubation with anti-CD99 antibody (1:3500) in antibody diluent for 1 h at 37 °C, then with secondary antibody as above, then with Opal 690 (1:100) for 10 min at RT. All slides were counterstained with DAPI (BioCat, Heidelberg, Germany) and enclosed with Prolong Diamond Antifade (Thermo Fisher). Imaging was performed with Vectra^®^ 3.0 system (Perkin Elmer, Rodgau, Germany). A whole slide scan was first acquired at 10× magnification, across the full emission light spectrum in each filter tube—DAPI (450–470 nm), FITC (505–545 nm), CY3 (580–620 nm), Texas Red (600–650 nm), and CY5 (670–720 nm). Four regions of each slide were chosen for multispectral imaging, using the image analysis software Phenochart™, and were re-scanned at 20× magnification. Multispectral images were unmixed using spectral libraries built from images of single-stained tonsil or placenta control tissues for each individual fluorophore, with the InForm Analysis Software (InForm 2.1.4, Akoya Bioscience). The autofluorescence was subtracted from unmixed multispectral images of an EwS tissue slide that had undergone the complete staining procedure, omitting the primary antibodies. The images were trained using the InForm software (tissue segmentation, cell segmentation, and phenotyping tools). The same algorithms were used to analyze HLA-G/HLA-E-positive and -negative tumor tissues. Data processing and analysis were performed with R software version 3.6.3 and integrated development environment RStudio version 1.4., with packages phenoptrR and phenoptr Reports (Akoya Bioscience).

**Statistical analysis.** Data were analyzed and visualized using IBM SPSS Statistics 27 for Windows and Systat SigmaPlot 11.0 software. Statistical analysis was performed with the paired *t*-test, unless otherwise indicated in the figure legends. Results were considered to be statistically significant at *p* ≤ 0.05.

## 3. Results

### 3.1. IFN-γ Cytokine Stimulated EwS Cells Express HLA-G Isoform HLA-G1

**Figure 1:**
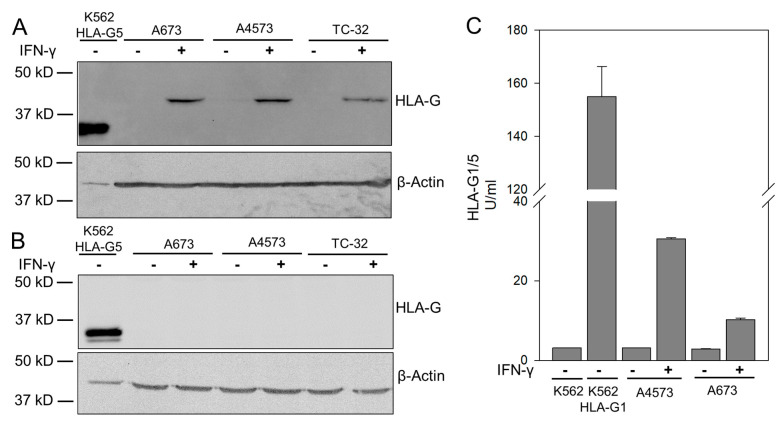
IFN-γ stimulated EwS cells express HLA-G isoform G1. (**A**,**B**) Western Blot analysis of HLA-G expression in whole cell lysates of HLA-G5-transduced K562 cells (positive control; a, 1 µg; b, 5 µg) and EwS cell lines A673, A4573 and TC-32, following pretreatment with IFN-γ or untreated (all 50 µg). (**A**) Anti-HLA-G antibody clone 4H84 (recognizing all isoforms) and (**B**) Anti-HLA-G antibody clone 5A6G7 (recognizing only isoforms HLA-G5 and G6). (**C**) Expression of HLA-G in whole cell lysates (25 µg) of wild-type K562 (negative control), HLA-G1 transduced K562 cells (positive control), and EwS cell lines A4573 and A673, following pretreatment with IFN-γ or untreated, using an ELISA exclusively detecting the isoforms HLA-G1 and G5.

To investigate the capacity of HLA-G to inhibit the antigen-specific effector functions of CART, we aimed to selectively disrupt the HLA-G gene in EwS cells through targeted mutagenesis. For this purpose, we first set out to identify the individual HLA-G isoforms expressed in EwS among a total of 4 membrane-bound (HLA-G1 to G4) and two soluble (HLA-G5 to -G7) isoforms generated by alternative splicing from the HLA-G transcript [23]. EwS cells were pretreated with IFN-γ to induce expression of HLA-G, as previously described [26]. First, we attempted to determine the amino acid sequence of HLA-G in whole cell lysates from IFN-γ-pretreated EwS cells using tandem mass spectrometry and high-performance liquid chromatography. However, the protein sequence similarities between non-classical and classical HLA molecules prevented fractionation of the low quantities of HLA-G expressed by EwS against the high-background of classical HLA molecules (Appendix A). Therefore, we used an indirect approach, based on isoform-specific antibodies, to identify HLA-G isoforms by Western Blot and by ELISA, along with K562 control cells gene-modified to express either HLA-G1 or HLA-G5. Monoclonal antibody clone 4H84 that binds all HLA-G isoforms detected protein bands corresponding in size to either HLA-G1 or -G5 in lysates from all 3 EwS cell lines (A673, TC-32, A4573) after IFN-γ stimulation, and in HLA-G5 transduced K562 cells (Figure 1a). In contrast, clone 5A6G7, which selectively binds HLA-G5 and -G6, failed to bind protein except in the HLA-G5-transduced control cell line (Figure 1b), suggesting that the isotype produced by EwS cells is G1. This result was reproduced by an HLA-G1/5 specific ELISA that detected HLA-G in lysates of both HLA-G1 transduced K562 control cells, as well as IFN-γ stimulated EwS cell lines A673 and A4573 (Figure 1c). We conclude that the HLA-G isoform induced in EwS by IFN-γ cytokine stimulation is HLA-G1. 

### 3.2. HLA-G Expression on EwS Cells Does Not Directly Impair Cytolysis by G_D2_-Specific CART

To study the functional significance of HLA-G for T cell responses against EwS cells, we aimed to selectively eliminate HLA-G in EwS through CRISPR/Cas9 genome editing. Specific single guide RNAs (sgRNAs) were generated against the α1 domain of HLA-G. High-sequence homologies to other HLA molecules did not allow generation of sgRNAs with target specificities of >20 by MIT specificity score. To disrupt the HLA-G gene, we lentivirally transduced A4573 cells with 6 different sgRNA candidates (Appendix B). Following up to 3 weeks of in vitro selection, HLA-G protein was still detected by Western Blot analysis in EwS cells transduced with either of all sgRNA candidates. To enhance the specificity of the knockout, we generated single cell clones from A4573 cells transduced with the sgRNA with the highest specificity score, sgRNA129, followed by further in vitro selection. PCR analysis of genomic DNA from 15 clones led to the identification of a single knock-out clone. Disruption of the HLA-G gene sequence was confirmed by sequencing analysis. Yet, residual HLA-G protein remained detectable by Western Blot analysis and ELISA (Appendix B). A potential explanation is heterozygosity of the gene knockout, with gene sequencing detecting the knockout allele. 

**Figure 2:**
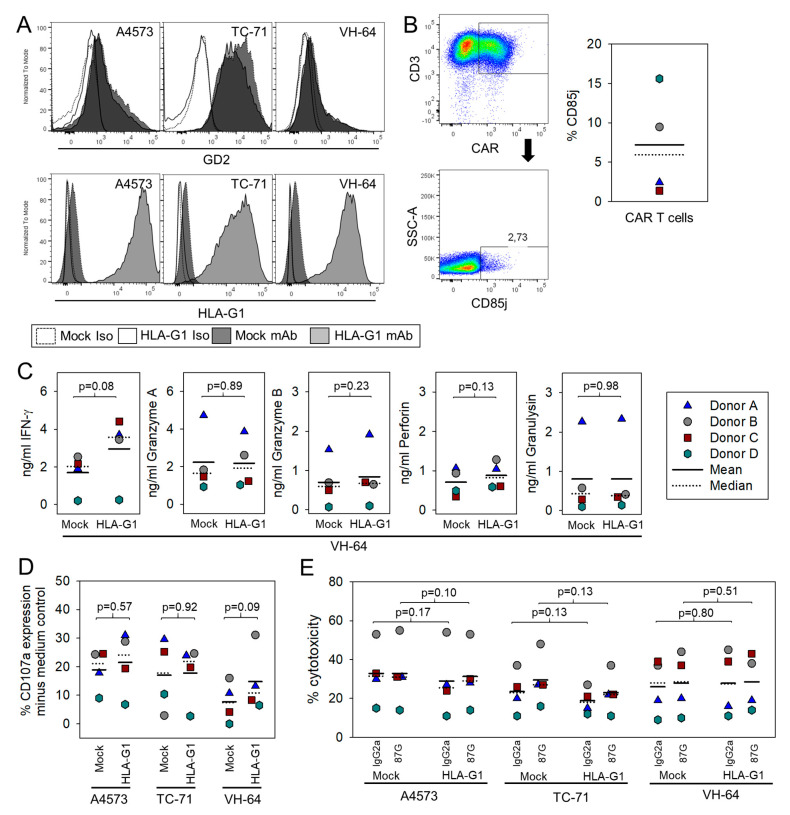
HLA-G1 expressed by EwS cells does not impair antigen-induced cytokine release or cytolysis by CAR gene-modified effector T cells. (**A**) HLA-G1 and G_D2_ expression on the cell surface of the G_D2_-positive EwS cell lines A4573, TC-71 and VH-64. (**B**) CD85j (ILT-2) surface expression in GD2-BBζ CART. (**C**) Secretion of IFN-γ and of perforin, granzymes A and B and granulysin by GD2-BBζ CART, upon antigen-specific activation with HLA-G1- transduced and mock-transduced G_D2_-positive EwS cells (VH-64). (**D**) CD107a upregulation by CD85j-expressing GD2.BBζ CAR-transduced T cells from 4 individual healthy donors, in response to coincubation with HLA-G1 transduced G_D2_-positive EwS cell lines. Mock-transduced EwS cells from the same cell lines (HLA-G1 neg) were used as controls. (**E**) Cytolysis of HLA-G1-transduced EwS cells from 3 G_D2_-positive cell lines by GD2.BBζ CAR-transduced T cells from 4 healthy donors, with and without preincubation (30 min) with HLA-G1-specific blocking antibody clone 87G (20 μg/mL) or IgG2a control (E:T Ratio 40:1). Mock-transduced EwS cell lines (HLA-G1-neg) were used as controls.

We concluded that the high sequence homology between the HLA-G gene and other HLA genes represents a significant barrier to successful targeted mutagenesis. Therefore, we switched to a knock-in model and overexpressed HLA-G1 in 3 G_D2_-positive EwS cell lines (A4573, TC-71, and VH-64) [28] through retroviral gene transfer (Figure 2a). HLA-G1 transduced EwS cells were used as targets for T cells gene-modified to express a G_D2_-specific 4-1BB costimulated second-generation CAR (GD2-BBζ). Surface expression of the HLA-G receptor CD85j (ILT (immunoglobulin-like transcript) 2) was detected in variable proportions of CAR-transduced T cells generated from 4 individual healthy donors (Figure 2b). No differences in the patterns and magnitude of secretion of cytokine IFN-γ or cytotoxic granules were found in response to HLA-G1-wildtype and HLA-G1-transduced EwS cells through multiplex analysis (Figure 2c). The presence of HLA-G1 on the cell surface of G_D2_-positive EwS cells did not significantly reduce the G_D2_-target-specific degranulation response of GD2-BBζ CART, even when gating on CD85j-positive CART (Figure 2d). HLA-G1 wildtype and HLA-G1-transduced tumor cells from the same EwS cell lines were lysed equally well by GD2-BBζ CART, regardless of the presence or absence of HLA-G1 blocking antibody 87G [29] (Figure 2e). Thus, HLA-G1 surface expression in EwS cells does not affect their ability to induce CART activation responses, nor their sensitivity to antigen-specific lysis by CAR gene-modified T cells.

### 3.3. HLA-G Expression on Myeloid Bystander Cells Reduces the Degranulation Response of G_D2_-Specific CART

After disproving a direct inhibitory interaction between the HLA-G-positive EwS cells and tumor-antigen specific CART, we addressed the hypothesis that myeloid bystander cells in the TME can mediate the T-cell inhibitory effects of HLA-G in this cancer. In inflammatory environments, macrophages and other myeloid cells engage HLA-G by the ILT4 receptor, which induces a switch of their functional phenotype from activating to regulatory [21,22].

**Figure 3:**
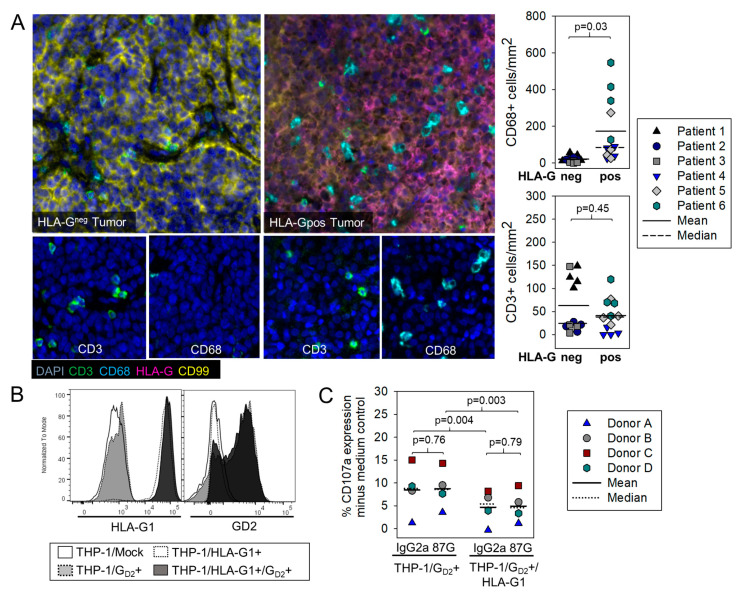
HLA-G1 expression in EwS is associated with the presence of myeloid cells, and HLA-G1 expressed by THP-1 monocytic cells reduces the degranulation response of CAR gene-modified effector T cells. (**A**) Representative multiplex immunofluorescence staining (20×) of HLA-G-negative (left panel) and HLA-G-positive (middle panel) human EwS biopsies with antibodies against CD3, CD68, HLA-G, CD99 and DAPI, as indicated. Scatter plot analysis of CD3+ T cell and CD68+ myeloid cell infiltration in HLA-G-positive and HLA-G-negative EwS biopsies (right panel). Immune cell densities (cells/mm^2^) were analyzed in 3 patients and in 4 areas per patient. We applied a generalized linear mixed model to compare HLA-negative and -positive patients with respect to CD68 and CD3 expression, respectively. Due to skewed distributions of expression values, a negative-binomial distribution with a log-link function was used. Repeated measurements per patient (i.e., areas) were modelled by a variance components-type covariance matrix. Wald-test *p*-values are reported for the group comparison. (**B**) HLA-G1 and G_D2_ coexpression on THP-1 cells after retroviral gene transduction with HLA-G1 and GD3S/GD2S genes. (**C**) CD107a expression of CD85j+ GD2.BBζ CAR-transduced T cells from 4 individual healthy donors, after coincubation with HLA-G1- and GD3S/GD2S-transduced THP-1 cells, with and without preincubation (30 min), with HLA-G1-specific blocking antibody clone 87G (20 μg/mL) or IgG2a control. THP-1 cells transduced only with the GD3S/GD2S transgene for G_D2_ target expression (HLA-G1-neg) were used as controls. Statistical analysis was performed with one-way repeated measures ANOVA.

To investigate whether HLA-G expression in EwS is associated with an inflammatory TME abundant with myeloid cells, we compared infiltration of CD3+ T cells and CD68+ macrophages in HLA-G-negative and HLA-G-positive pretreatment EwS biopsies through multicolor immunofluorescence staining (Figure 3a). HLA-G-positive EwS indeed had significantly higher proportions of infiltrating CD68+ myeloid cells as compared to HLA-G-negative biopsies, whereas the proportions of CD3+ T cells were comparable (Figure 3a).

To understand whether HLA-G1 expressed by myeloid cell populations can negatively affect antigen-specific effector responses of CART, we generated a model cell line coexpressing HLA-G1 along with surface G_D2_, by transducing the myeloid cell line THP-1 with genes encoding HLA-G1 as well as two key enzymes of glycolipid G_D2_ synthesis, GD2S and GD3S, which results in surface expression of G_D2_. Coexpression of HLA-G1 and G_D2_ in THP-1 cells after genetic modification was confirmed by flow cytometry analysis (Figure 3b). The presence of HLA-G1 on the cell surface of G_D2_-positive THP-1 cells significantly reduced G_D2_-target specific degranulation responses by GD2-BBζ CART from 4 individual donors (Figure 3c). Addition of HLA-G1 antibody 87G, described as blocking antibody [29], failed to fully restore antigen-specific degranulation of CART, in response to target cells. We conclude that negative effects on immune effector cells in the TME of HLA-G-positive tumors can be mediated by myeloid bystander cells rather than by direct interactions between HLA-G expressing tumor cells and CART. HLA-G antibody clone 87G in our experimental system was inadequate to fully reverse the inhibitory effect on CART.

### 3.4. HLA-E Is Expressed in EwS and Associated with Infiltrating T Cells Both in Human Pretherapeutic Biopsies and in Murine EwS Xenografts Treated with GD2.BBζ CART In Vivo, and CART Recovered from Treated Mice Express the HLA-E Receptor NKG2A

**Figure 4:**
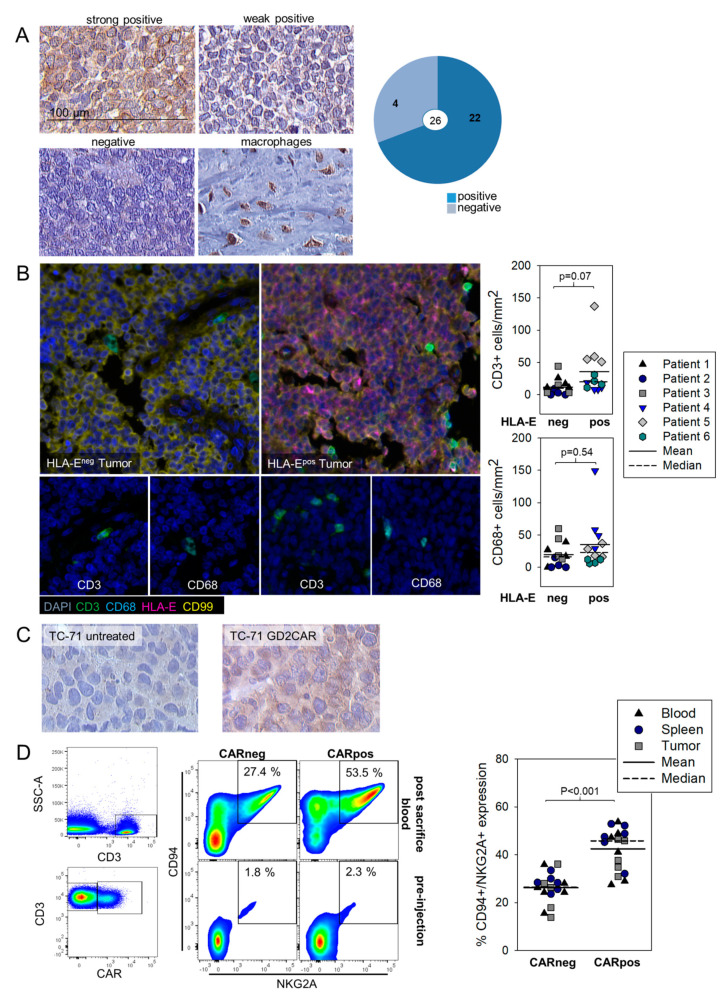
HLA-E is expressed in EwS cells both in vitro and in vivo and associated with T cell infiltrations. (**A**) HLA-E expression by immunohistochemistry analysis of 26 EwS biopsies. Shown is one example each for strong, weak, and lack of HLA-E expression, an example for HLA-E expressed on tumor-infiltrating macrophages and a summary of the results. (**B**) Representative multicolor immunofluorescence staining (20×) of HLA-E-negative (left panel) and HLA-E -positive (middle panel) EwS biopsies with antibodies against CD3, CD68, HLA-E, CD99 and DAPI, as indicated. Scatter plot analysis of CD3+ T cell and CD68+ myeloid cell infiltration in HLA-E-negative and HLA-E-positive EwS biopsies (right panel. Immune cell densities (cells/mm^2^) were analyzed in 3 patients and in 4 areas per patient. We applied a generalized linear mixed model to compare HLA-negative and -positive patients with respect to CD68 and CD3 expression, respectively. Due to skewed distributions of expression values, a negative-binomial distribution with log-link function was used. Repeated measurements per patient (i.e., areas) were modeled by a variance components-type covariance matrix. Wald-test *p*-values are reported for the group comparison. (**C**) HLA-E expression in TC-71 EwS xenografts following in vivo treatment with GD2-BBζ CART. (**D**) CD94 and NKG2A expression of CART after 28 days in a TC-71 EwS xenograft model.

A related non-classical HLA molecule commonly overexpressed on the surface of various cancers is HLA-E. Interactions of HLA-E with NKG2A on T cells and NK cells was found to restrain cytotoxic anti-tumor effector functions [19,20], and a humanized monoclonal antibody interfering with this interaction can act as checkpoint inhibitor to rescue T cell functions against tumors, in a synergistic manner with blockade of the established PD-1 checkpoint [18]. To investigate a potential role of HLA-E in the immune defense of EwS, we stained pretherapeutic tumor biopsies from 26 patients with anti-HLA-E antibodies by immunohistochemistry. The majority (22/26, 85%) of EwS samples expressed HLA-E on tumor cells, infiltrating macrophages or both (Figure 4a).

Comparisons of immune cell infiltrates in HLA-E positive and negative EwS tumor samples through multicolor immunofluorescence staining (Figure 4b) did not reveal significant associations of HLA-E expression with higher densities of infiltrating myeloid cells or T cells. To understand whether infiltrating antitumor T cells can induce HLA-E expression in EwS, we analyzed HLA-E in s.c. TC-71 EwS xenografts from mice treated with GD2.BBζ CART (Appendix C). Indeed, we found HLA-E expressed in xenografts following adoptive transfer in vivo of GD2.BBζ-transduced T cells, but not in control tumors from untreated mice (Figure 4c). Peripheral blood, spleen, and tumor-infiltrating T cells obtained from CART-treated mice on day 28 of the experiment were analyzed by flow cytometry for expression of the HLA-E receptor NKG2A. Gating on CAR-positive and CAR-negative T cell populations revealed a significant association and upregulation of NKG2A with T cells expressing the CAR (Figure 4d).

Thus, HLA-E is common in EwS and is associated with T cell infiltration, both in human patients and in murine xenografts treated with tumor-antigen specific CART. Through expression of NKG2A, CART can engage HLA-E.

### 3.5. HLA-E Does Not Impair Degranulation Responses against EwS Cells or Myeloid Cells by G_D2_-Specific CART

**Figure 5:**
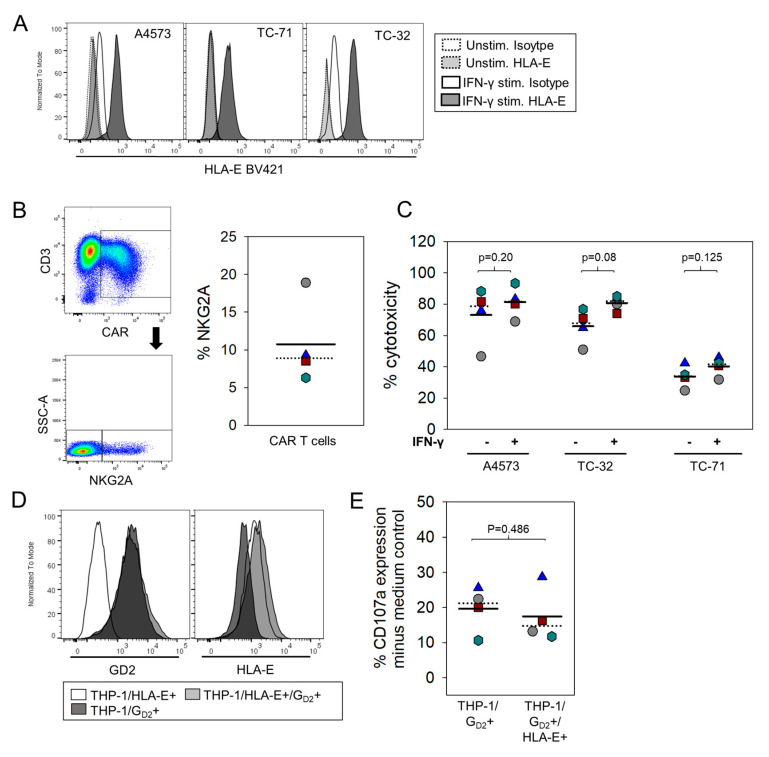
HLA-E expressed by THP-1 monocytic cells does not affect functional responses of CART. (**A**) Upregulation of HLA-E in EwS cells after incubation with IFN-γ. (**B**) NKG2A expression by flow cytometry in GD2-BBζ CART generated from 4 donors. (**C**) Cytotoxicity assay of IFN-γ-induced, HLA-E-expressing EwS cells as targets for GD2-BBζ-transduced effector T cells from 4 donors (effector-to-target ratio 40:1). (**D**) HLA-E and G_D2_ coexpression on THP-1 cells after retroviral gene transduction with HLA-E and GD3S/GD2S genes. (**E**) CD107a expression of GD2.BBζ CART from 4 individual healthy donors after coincubation with HLA-E- and GD3S/GD2S-cotransduced THP-1 cells, and with HLA-E-negative, GD3S/GD2S-transduced THP-1 cells as controls. CD107a expression was analyzed after gating on CD3+ NKG2A+ CART.

Finally, we addressed the question whether HLA-E expression in EwS directly or indirectly via myeloid cells affects the functional antitumor responses of tumor-antigen specific CART. As in vitro model, we used EwS cells pretreated with IFN-γ, which reliably induces expression of HLA-E on the cell surface (Figure 5a), and GD2.BBζ CART expressing NKG2A at variable proportions (Figure 5b). In a direct cytotoxicity assay, IFN-γ-pretreated EwS cells were equally sensitive to lysis by G_D2_-specific-CART as compared to non-pretreated, HLA-E-negative EwS cells (Figure 5c). Thus, HLA-E does not impair the cytolytic capacity of tumor-antigen-specific CART in direct interactions with EwS cells. To investigate the capacity of the myeloid cell line THP-1 to impair CART degranulation responses via HLA-E, we coexpressed HLA-E and G_D2_ in THP-1 cells through gene transfer of HLA-E and GD3S/GD2S (Figure 5d) and used these cells as target for GD2-BBζ CART. Degranulation responses of CART did not significantly vary with and without HLA-E expressed on THP-1 cells (Figure 5e). Together, these data argue against a relevant inhibitory effect of the HLA-E/NKG2A checkpoint, in CART therapy of EwS.

## 4. Discussion

In view of novel cellular cancer therapies, identification of molecules in the TME that compromise the function of T cells is gaining importance. While antagonists of inhibitory checkpoints alone are ineffective in cancers lacking tumor-infiltrating T cells with native specificity for cancer-associated neoantigens, they could serve to enhance the activity and persistence of adoptively transferred CAR-engineered T cells, to create powerful combination strategies [30]. With their physiological roles in immune tolerance and expression in many human solid cancers, HLA-G and HLA-E are both candidate targets for novel immune checkpoint inhibitors [18,19,20,21,22,23,24,25].

In addition to our previous work [6,26], we provide new evidence for an association of HLA-G and now also HLA-E expression with EwS: Immunohistochemistry analysis of pretherapeutic tumor biopsies detected HLA-E at variable densities in 85% of 26 EwS patients (Figure 4a), compared to 66% of HLA-G positive tumors among 59 EwS patients in a previous report [26]. Comparable to HLA-G, HLA-E can be expressed on tumor cells, stroma cells or both. Both molecules are upregulated under inflammatory conditions in this cancer, mimicked by IFN-γ stimulation in vitro (Figure 1 and Figure 5a) and by treatment of xenograft tumors with G_D2_-specific CART in vivo [6,26] (Figure 4c). Thus, HLA-G and HLA-E are consistent features of EwS and are responsive to inflammatory stimuli in this cancer. Considering their immune-inhibitory properties, these molecules constitute a theoretical challenge for the optimal activity of tumor-antigen specific CART.

Contrary to expectations, the work presented here strongly argues against a direct negative effect of HLA-G and HLA-E immune checkpoints on CART in EwS. Neither HLA-G1 nor HLA-E had any functional significance for the target-induced in vitro responses of CART against EwS cells, when artificially overexpressed on tumor cells (Figure 2 and Figure 5). This included various parameters of target-induced T cell activation, such as cytokine secretion, upregulation of the degranulation marker CD107a and direct target cytolysis. Thus, CART at least in vitro are remarkably resistant against the inhibitory action of immunosuppressive HLA antigens.

One explanation could be the low and variable expression of inhibitory receptors on G_D2_-specific CART in vitro, which directly engage HLA-G (CD85j, Figure 2b) or HLA-E (NKG2A, Figure 4d), respectively. This finding is in line with published reports of a high variability of CD85j and NKG2A expression on human CD3+ and CD8+ T cells affected by donor age, T cell source, antigen experience, and also technically by individual antibody clones used for surface detection [31,32,33,34]. Activating cytokines and inflammatory conditions in infections and cancer are known to increase the proportions of NKG2A or CD85j-expressing T cells [18,35,36,37,38]. Own work by our group found that exposure of G_D2_-specific CART-expressing NK cells to G_D2_-positive EwS cells induces upregulation of CD85j on NK cells [6]. In vitro retroviral engineering of human T cells with CAR genes, despite involving CD3- and CD28-mediated in vitro T cell activation and IL-2 cytokine expansion, did not produce higher populations of T cells expressing CD85j (Figure 2b) or NKG2A (Figure 4d). However, ex vivo studies in our xenograft model revealed substantial upregulation of NKG2A on CART, following G_D2_-specific CART therapy (Figure 4d). Thus, with all the limitations of the murine xenograft model, CART can upregulate NKG2A upon antigen encounters. However, since even the selective analysis of CD85j or NKG2A-positive subpopulations of CART failed to identify any inhibitory effect of HLA-G or HLA-E expressing EwS targets on the strength of the cytolytic response (Figure 2d and Figure 5e), the functional relevance of engagement of these receptors on CART remains questionable.

Another explanation for the unexpected HLA-G/HLA-E resistance of CART is that relevant HLA-G or HLA-E-mediated immune-inhibitory effects could rely not on direct interactions with CART but on bystander cells in the TME, a scenario not reflected by coculture experiments of CART with HLA-G/HLA-E expressing tumor cells. EwS is characterized by high densities of tumor-associated macrophages [39] (Figure 3a and Figure 4b), and myeloid cell infiltration has been associated with lack of in vivo activity of CART in murine models of this cancer [8], suggesting important roles of myeloid cell populations in immune escape. Indeed, we found HLA-G expression to be associated with infiltrating myeloid cells in pretherapeutic EwS biopsies (Figure 3a). In support of this hypothesis, HLA-G did have a functional impact on antigen-specific CART responses when artificially expressed on a monocytic cell, rather than on the tumor cell itself (Figure 3c). Thus, HLA-G could still be a potent functional barrier to CART targeting when expressed in the context of myeloid bystander cells, as is frequently the case in EwS, by triggering additional inhibitory pathways. At the same time, HLA-G expressed by EwS cells could engage the receptor ILT4 on tumor-associated macrophages, resulting in acquisition of immune-suppressive properties and selective expansion of myeloid suppressor cells [16,40]. Thereby, HLA-G could contribute to a hostile TME and to local immune escape of EwS from CART-mediated immune control. The association with infiltrating myeloid cells in human EwS biopsies was limited to HLA-G and not found with HLA-E (Figure 4b). Accordingly, HLA-E expressed by a monocytic cell type failed to affect CART responses (Figure 5d,e). This argues against but does not rule out indirect bystander effects of HLA-E on CART function. In vivo studies reflecting the complexity of HLA-G and HLA-E checkpoints by acknowledging indirect interactions between EwS cells and CART via myeloid cells are impeded by the lack of in vivo EwS models that adequately reflect these interactions within a single species. Meaningful in vivo studies will have to be performed in humanized mouse models. As a third explanation, non-classical HLA molecules in EwS could be indicators of inflammatory components of the TME, without making any active contributions to immune inhibition. While a vast number of reports exist that associate either HLA-G or HLA-E with relapse and poor clinical outcome [19,25,41,42,43,44], evidence for the capacity of these molecules to suppress antitumor responses by CD8+ T cells remains limited. To our knowledge, this is the first report addressing the potency of either HLA-G or HLA-E to impair CART-mediated antitumor responses. A recently developed antagonist of the HLA-E/NKG2A interaction, monalizumab, was found to rescue NK cell activity against tumors as stand-alone therapy, but required concomitant PD-1 checkpoint blockade for enhancing efficacy of antigen-specific T cells [18]. Thus, HLA-G or HLA-E could still be relevant inhibitors of CART function in EwS in the context of additional inhibitory or tolerogenic pathways. More extensive studies of candidate immune checkpoints in EwS, besides PD-L1, including alternative ligands of the B7 family and others, e.g., the Nectin-like family, could be helpful to understand in more detail how this cancer affects immune effector cells and their function.

Finally, technical limitations could have affected the outcome of the functional experiments. Compared to the knock-in studies that we performed by artificially expressing HLA-G and HLA-E in EwS cells, genetic knockout could have revealed more subtle functional roles. In our hands, the high sequence homology of HLA-G with other HLA molecules did not allow us to perform a specific genetic knockout. The difficulty to discern HLA-G isoforms against the background of classical HLA molecules not only affected identification of relevant isoforms but could also limit the development of therapeutic blocking antibodies.

This work was motivated by the urgent medical need to develop more effective therapies against EwS, which remains a fatal malignancy in many patients despite highly intensive multimodal treatment strategies. Adoptive transfer of CAR-engineered T cells overcomes the lack of effector cells in the immunological desert of EwS, which explains the failure of PD-1 checkpoint antagonists to produce clinical responses. Yet, T-cell based immunotherapies will need to be combined with agents that enable optimal effector functions in the context of the solid tumor microenvironment. HLA-G or HLA-E could be valuable enhancers of immune responses in other cancers and for NK-cell targeted strategies, as shown for the anti-NKG2A blocking antibody monalizumab now developed as a novel first-in-class checkpoint inhibitor for HLA-E-positive cancers [18]. For EwS, the search continues for strategies that overcome locoregional immune escape in the tumor niche.

## 5. Conclusions

While HLA-G and HLA-E expression is common in EwS, with consistent upregulation under inflammatory conditions, these checkpoints do not affect the function of G_D2_-specific CART, unless HLA-G is expressed on myeloid bystander cells. The functional robustness of CART against HLA-G and HLA-E expressed on EwS cells argues against the use of inhibitors of these molecules to enhance CART-mediated antitumor responses. Yet, non-tumor cells of myeloid origin are widely present in EwS and likely to contribute to an immune-suppressive TME in this cancer. Therefore, our observation of a potential role of HLA-G in mediating inhibitory bystander effects on CART deserves further investigation. 

Effective CART-targeting of EwS has multiple requirements. CART must infiltrate into sarcoma tissues and exert potent cytolytic effector functions against tumor cells, while acquiring a memory T cell state that allows the cells to persist and become reactivated. Any single-targeted checkpoint inhibitors will likely be insufficient to bring CART to their full potential in this and other solid cancers. More promising are functional enhancers that simultaneously co-target various impeding mechanisms. One strategy could be a target-induced release of a multifunctional cytokine that counteracts immune-suppressive pathways while enhancing the activity and persistence of CART and recruiting additional effector cells [45].

## Figures and Tables

**Figure A1:**
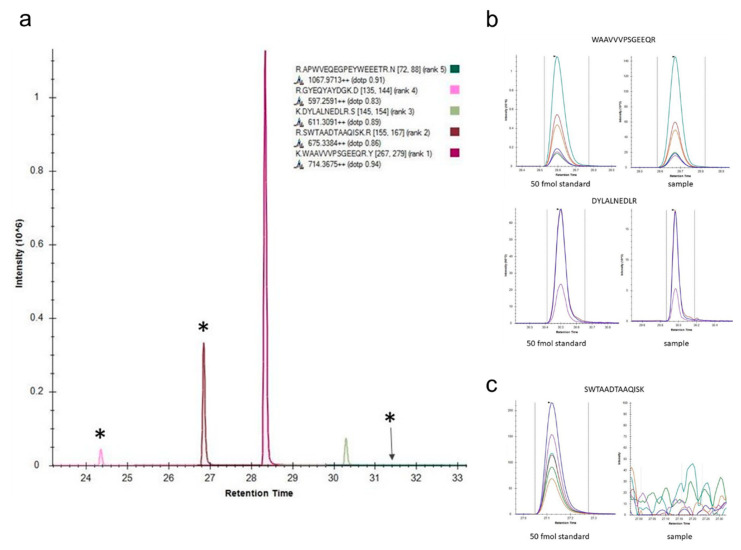
(**a**) Target peptide ion response in the pseudo multiple reaction monitoring experiment (for details see [46]). One peptide is identical in six other proteins and is used as a background marker (WAAVVVPSGEEQR). Another peptide (DYLALNEDLR) partially serves the same purpose. It differs in two amino acids, but the change from leucine to isoleucine cannot be distinguished by mass spectrometry in five other human proteins as these amino acids have the same residue mass. The starred peaks correspond to the unique peptides of HLA-G. (**b**) Response of peptides WAAVVVPSGEEQR and DYLALNEDLR in reference and an exemplary sample. (**c**) Response of unique peptide SWTAADTAAQISK in reference and an exemplary sample.

**Figure A2:**
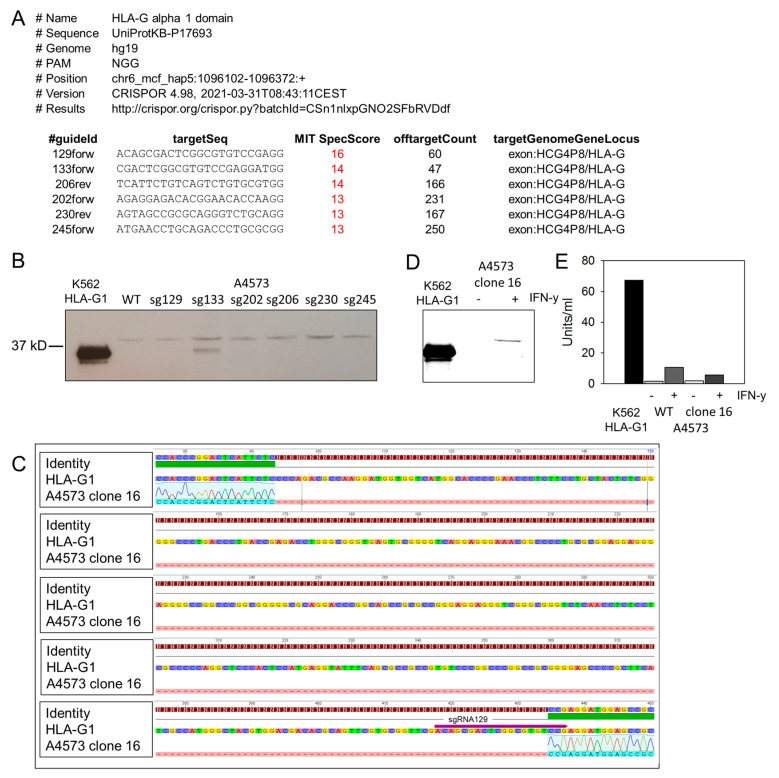
CRISPR/Cas9 knock-out. (**A**) Design of single guide RNA sequences. (**B**) Western Blot analysis of HLA-G expression in whole cell lysates of HLA-G1-transduced K562 cells (positive control) and the CRISPR/Cas9-sgRNA-transduced EwS cell line A4573, following pretreatment with IFN-γ. Wildtype A4573 cells were used as a control. (**C**) Genomic DNA sequence analysis of CRISPR/Cas9-sgRNA 129-transduced EwS cell line A4573 after single cell cloning. (**D**) Western Blot analysis of HLA-G expression in whole cell lysates of HLA-G1-transduced K562 cells (positive control) and the CRISPR/Cas9-sgRNA 129-transduced EwS cell line A4573 after single cell cloning, following pretreatment with IFN-γ or without. (**E**) Expression of HLA-G in whole cell lysates of HLA-G1 transduced K562 cells (positive control) and the CRISPR/Cas9-sgRNA 129-transduced EwS cell line A4573 after single cell cloning, following pretreatment with IFN-γ or untreated, using an ELISA for the isoforms HLA-G1 and G5. Wildtype A4573 cells were used as a control.

**Figure A3:**
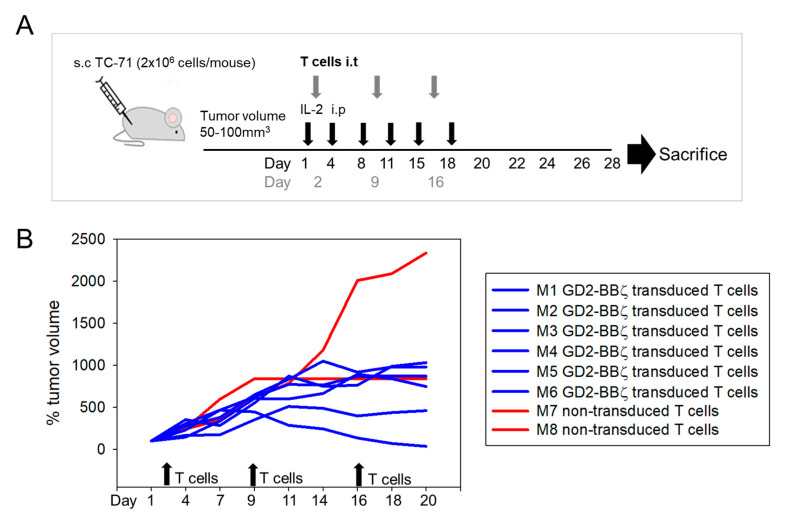
In vivo experiment used to provide tumor samples for HLA-E analysis. (**A**) Schematic of the in vivo experiment and timeline. (**B**) Percent (%) tumor volume. Murine blood, spleen, and EwS xenografts were obtained from previous mouse experiments approved by the animal care committee of the local government (LANUV, Recklinghausen, Az. 84-02.04.2014.A160). TC-71 cells had been injected subcutaneously into 8–12 week-old NOD scid gamma (NSG) mice. Upon a tumor volume of 50–100 mm^3^, 3 doses of 5 × 10^6^ gene-modified, GD2.BBζ T cells were injected intratumorally over a period of 2 weeks. Control mice received analogous injections of non-transduced T cells. Mice were sacrificed either earlier according to the score sheets or after a maximum of 28 days, post tumor injection. The analytic endpoint was relative tumor growth (in %), and was determined by dividing the tumor volume (mm^3^) at the time of analysis by the initial tumor volume on day 1 (mm^3^) (×100). For histological analysis, tumor tissue had been preserved. The experiment was not designed and performed for the direct purpose of the current research question. Outcome analysis of this in vivo experiment is not a subject of the current report.

## Data Availability

The data that support the findings of this study are available from the corresponding author upon reasonable request.

## Supplementary Materials

The following are available online at https://www.mdpi.com/article/10.3390/cancers13122857/s1: Figure S1: The original western blotting figures.

## Appendix A

Mass spectrometric quantification of human HLA-G was attempted as described [46]. Five peptides were selected for target analysis. Not many peptides were suited as markers. In fact, the most abundant peptide (WAAVVVPSGEEQR) is homologous in at least six different human proteins, another peptide (DYLALNEDLR) in at least five. They were still used in the analysis, next to three truly unique peptides for HLA-G as a background check. Figure A1 shows the respective peptide ion response in the experiment.

In the samples, peptides WAAVVVPSGEEQR and DYLALNEDLR were detected with confidence and good signal strength (Figure A2), in contrast to the unique peptides of HLA-G. They were not seen properly, as is exemplarily shown in Figure A3.
