# Supplementary material for: HLA-G and HLA-E Immune Checkpoints Are Widely Expressed in Ewing Sarcoma but Have Limited Functional Impact on the Effector Functions of Antigen-Specific CAR T Cells
Source: Cancers (Basel). 2021 Jun 8;13(12):2857. doi: 10.3390/cancers13122857 (PMC8227123; doi:10.3390/cancers13122857)
Supplement: Supplementary file 1 [file cancers-13-02857-s001.zip › cancers-1233142-supplementary.pdf]

Figure S1A

Western Blot HLA-G clone 4H84

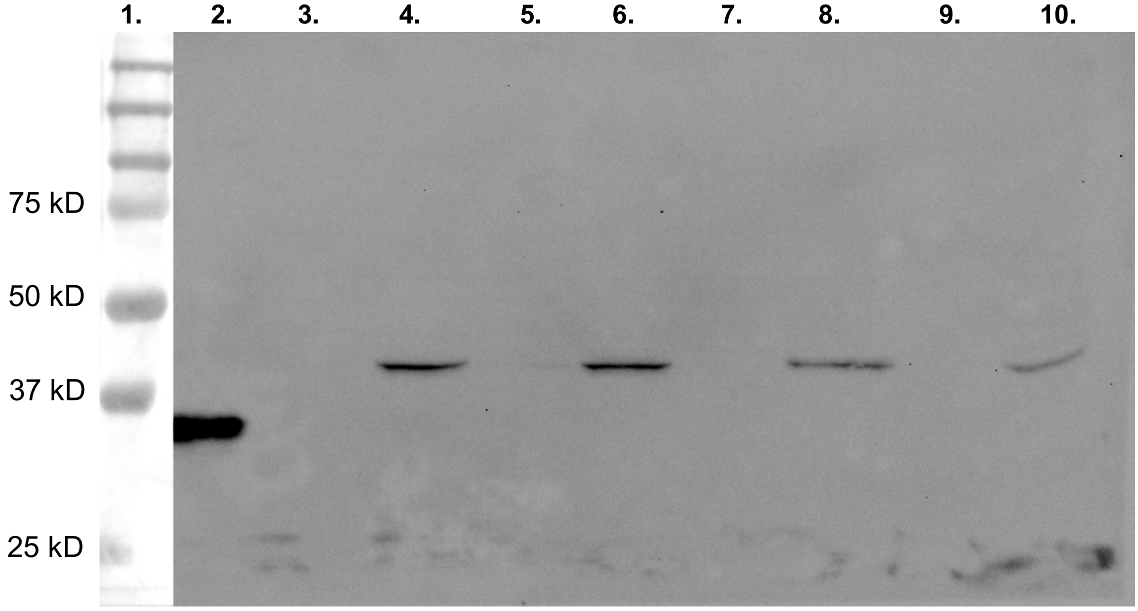

Western Blot β-Actin

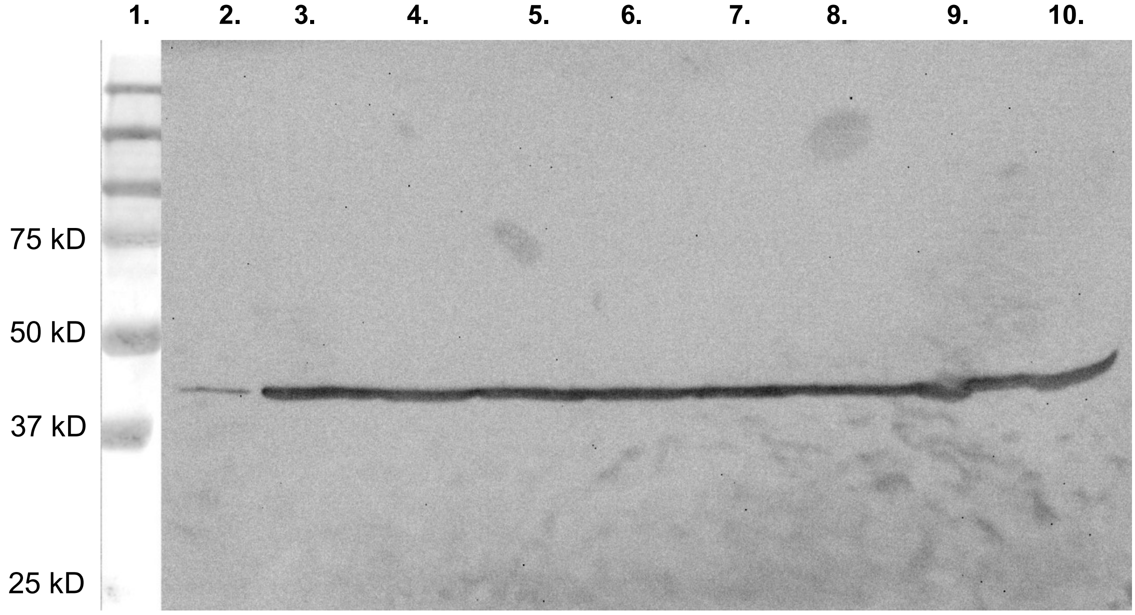

1. Marker
2. K562-HLAG5; 1 µg
3. A673 unstimulated; 50 µg
4. A673 IFNγ 2000U 48h; 50 µg
5. A4573 unstimulated; 50 µg
6. A4573 IFNγ 2000U 48h; 50 µg
7. TC-32 unstimulated; 50 µg
8. TC-32 IFNγ 2000U 48h; 50 µg
9. MS-EWS-6 unstimulated; 50 µg
10. MS-EWS-6 IFNγ 2000U 48h; 50 µg

Figure S1B

Western Blot HLA-G clone 5A6G7

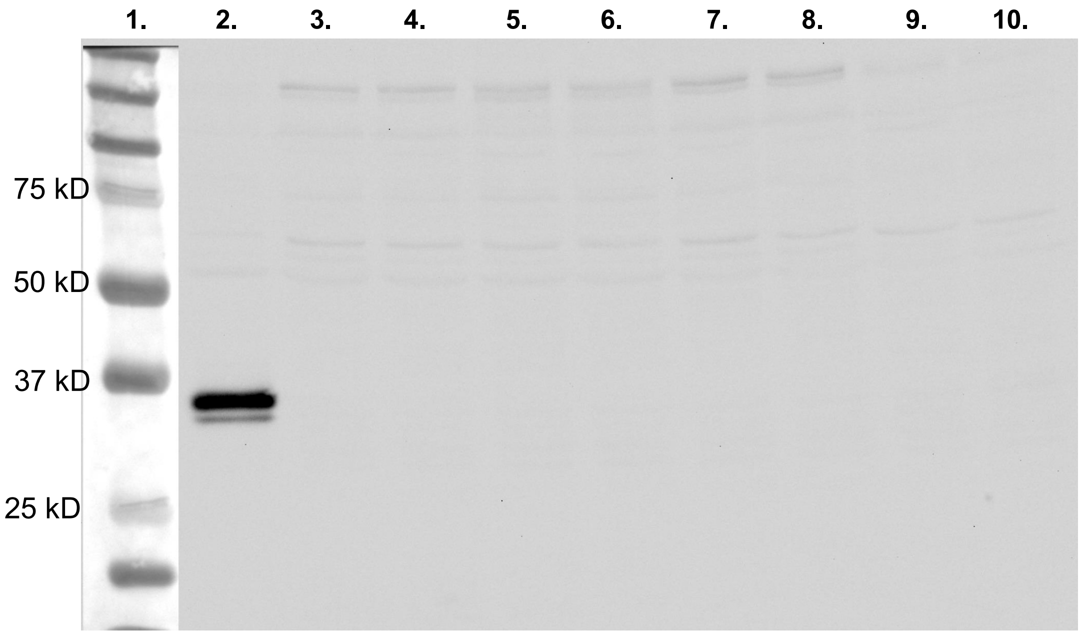

Western Blot β-Actin

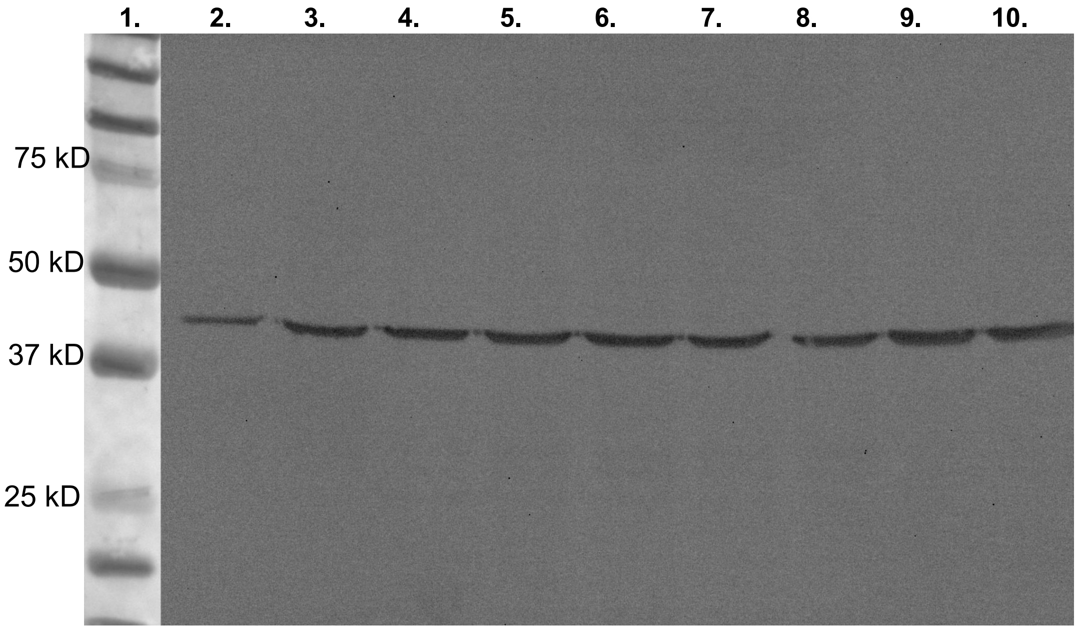

1. Marker
2. K562-HLAG5; 5 µg
3. A673 unstimulated; 50 µg
4. A673 IFNγ 2000U 48h; 50 µg
5. A4573 unstimulated; 50 µg
6. A4573 IFNγ 2000U 48h; 50 µg
7. TC-32 unstimulated; 50 µg
8. TC-32 IFNγ 2000U 48h; 50 µg
9. MS-EWS-6 unstimulated; 50 µg
10. MS-EWS-6 IFNγ 2000U 48h; 50 µg

Appendix B/B

Western Blot HLA-G clone 4H84

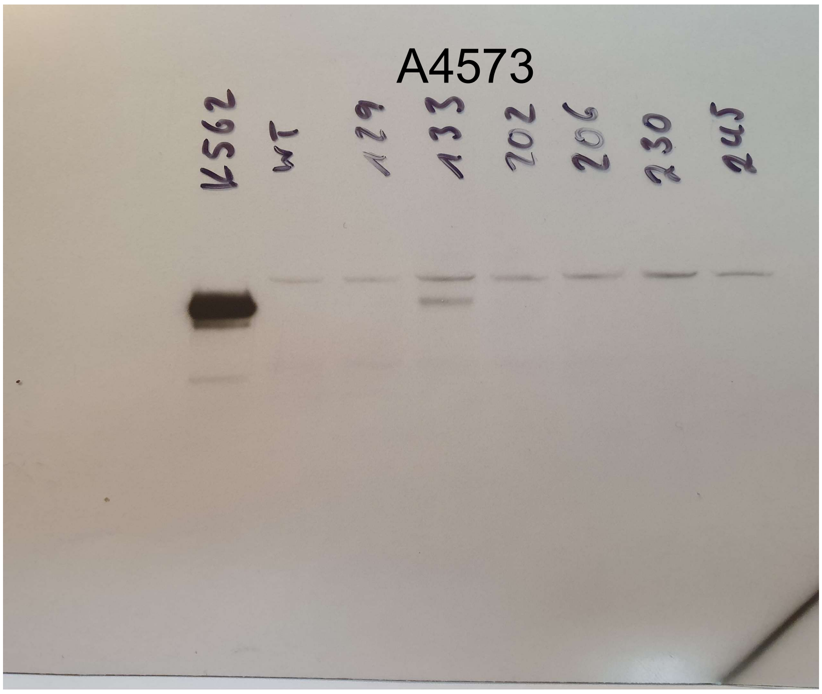

Appendix B/C

Western Blot HLA-G clone 4H84

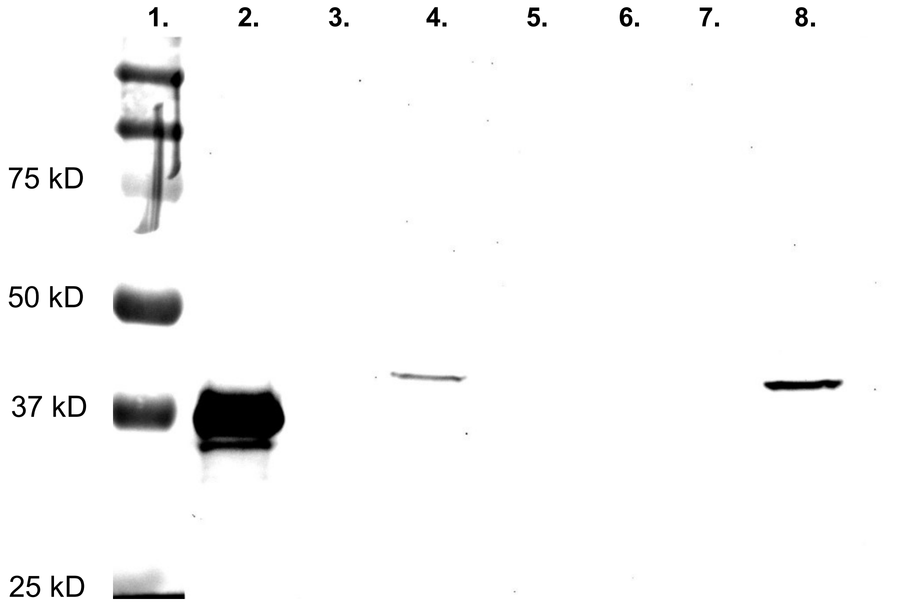

1. Marker
2. K562-HLAG1 1µg
3. A4573 K16 unstimulated
4. A4573 K16 IFNγ 2000U 48h
5. empty
6. empty
7. TC-32 unstimulated
8. TC-32 IFNγ 2000U 48h
